# Increased Age, but Not Parity Predisposes to Higher Bacteriuria Burdens Due to *Streptococcus* Urinary Tract Infection and Influences Bladder Cytokine Responses, Which Develop Independent of Tissue Bacterial Loads

**DOI:** 10.1371/journal.pone.0167732

**Published:** 2016-12-09

**Authors:** Matthew J. Sullivan, Alison J. Carey, Sophie Y. Leclercq, Chee K. Tan, Glen C. Ulett

**Affiliations:** 1 School of Medical Science, and Menzies Health Institute Queensland, Griffith University, Gold Coast, Queensland, Australia; 2 Research and Development Center, Ezequiel Dias Foundation (Funed), Belo Horizonte, MG, Brazil; Cedars-Sinai Medical Center, UNITED STATES

## Abstract

*Streptococcus agalactiae* causes urinary tract infection (UTI) in pregnant adults, non-pregnant adults, immune-compromised individuals and the elderly. The pathogenesis of *S*. *agalactiae* UTI in distinct patient populations is poorly understood. In this study, we used murine models of UTI incorporating young mice, aged and dam mice to show that uropathogenic *S*. *agalactiae* causes bacteriuria at significantly higher levels in aged mice compared to young mice and this occurs coincident with equivalent levels of bladder tissue colonisation at 24 h post-infection (p.i.). In addition, aged mice exhibited significantly higher bacteriuria burdens at 48 h compared to young mice, confirming a divergent pattern of bacterial colonization in the urinary tract of aged and young mice. Multiparous mice, in contrast, exhibited significantly lower urinary titres of *S*. *agalactiae* compared to age-matched nulliparous mice suggesting that parity enhances the ability of the host to control *S*. *agalactiae* bacteriuria. Additionally, we show that both age and parity alter the expression levels of several key regulatory and pro-inflammatory cytokines, which are known to be important the immune response to UTI, including Interleukin (IL)-1β, IL-12(p40), and Monocyte Chemoattractant Protein-1 (MCP-1). Finally, we demonstrate that other cytokines, including IL-17 are induced significantly in the *S*. *agalactiae*-infected bladder regardless of age and parity status. Collectively, these findings show that the host environment plays an important role in influencing the severity of *S*. *agalactiae* UTI; infection dynamics, particularly in the context of bacteriuria, depend on age and parity, which also affect the nature of innate immune responses to infection.

## Introduction

Urinary tract infections (UTIs) are among the most common bacterial infections affecting 150 million people each year worldwide [1]. UTIs are especially prevalent in women; one-third of all women will have experienced a UTI by the age of twenty-four and, with a risk of recurrent infection approaching 30% [2, 3], these infections account for a massive economic cost to the health care sector. Each infectious episode causes serious deterioration in the quality of life [4]. The majority of UTIs (~80%) are caused by *Escherichia coli*. A range of other bacteria including *Streptococcus agalactiae* can also efficiently colonise the genitourinary tract and cause various conditions resulting from UTI including cystitis, pyelonephritis and asymptomatic bacteriuria (ABU) [5–7]. *S*. *agalactiae* bacteriuria is especially important in pregnant women because infection has been associated with chorioamnionitis [8] and because of the potential for vertical transmission and neonatal disease [9]. Overall, the burden of *S*. *agalactiae* UTI in the United States is approximately 160,000 cases annually [7, 10]. Several studies have demonstrated that the pathogenesis of *S*. *agalactiae* UTI encompasses binding of the bacteria to the bladder [11, 12], cytotoxicity in urothelial cells, and elicitation of pro- and anti-inflammatory cytokine and chemokine responses [13, 14], as well as growth of some ABU-causing *S*. *agalactiae* in human urine that may affect persistence [15].

While globally it is estimated that *S*. *agalactiae* is associated with 2–3% of all UTIs [16, 17], the incidence of infection appears to differ among distinct patient populations such as healthy adults, pregnant women, and institutionalized elderly individuals [10, 18]. Reported prevalence rates of *S*. *agalactiae* ABU and UTI of 26% and 39% among diabetic gravidas women [19] and nursing home residents >70 years of age [20], respectively, for example, compare to rates of below 5.5% reported among healthy adult women [18]. These broad differences in *S*. *agalactiae* prevalence rates among distinct patient groups reported in epidemiological studies of UTI might reflect distinct elements of host susceptibility and pathogenesis in dissimilar host environments; prior studies, for example, have demonstrated pathogen-specific host responses [12] and immune modulation during *S*. *agalactiae* UTI [21] using experimental infection of mice to model human UTI [22]. Additionally, a multitude of recent studies have described host-driven determinants of the severity of UTI due to *E*. *coli*, as reviewed elsewhere [23]. However, the role of diverse host environments in affecting the pathogenesis of *S*. *agalactiae* UTI remains largely understudied. In this context, one recent study examined age and parity in *S*. *agalactiae* UTI and reported that parity increases bacterial colonisation of the bladder in multiparous mice compared to nulliparous mice [24]. *S*. *agalactiae* bladder colonisation in mice, reported in [24], was also found to be higher in young mice (8–10 weeks) compared to aged mice (7–11 months) [24], implying that younger age may predispose to *S*. *agalactiae* UTI; however, the study did not analyse cytokine responses nor assess levels of bacteriuria in the model.

Here, we sought to compare the dynamics of infection during *S*. *agalactiae* UTI using murine models incorporating distinct groups of mice to model states of young and old age, and nulliparous vs. multiparous backgrounds. We examined infection in the bladder, as reported previously [24], and bacteriuria and inflammatory mediators in the bladder that might influence UTI, but which have not been examined previously.

## Materials and Methods

### Bacteria

The serotype III uropathogenic *S*. *agalactiae* (UPSA) strain (0247) used in this study was previously described [12]. Briefly, the UPSA 0247 strain was originally cultured from clean-catch voided urine of a 35-year-old non-diabetic woman who presented to the University of Alabama Birmingham (UAB) Hospital with symptoms consistent with uncomplicated cystitis, including dysuria, single organism bacteriuria (>100,000 colony forming units [cfu] ml^-1^), and leukocyte esterase and pyuria (>10 white blood cells μl^-1^; non-spun), noted on urinalysis. Use of *S*. *agalactiae* cultured from the patient described in [12] was enacted according to the principles of the Helsinki Declaration with approval from the UAB Institutional Review Board (IRB) (X070722011) and the Human Ethics Committee (HEC) of Griffith University (MSC/11/10/HREC). The UAB IRB and the Griffith University HEC waived the need for specific informed consent. Bacteria were grown at 37°C on Todd-Hewitt (TH) agar or in TH broth (*Thermo Fisher Scientific*) agitated at 200 rpm for 16 h.

### Mice and murine model of UTI

Three different groups of female C57BL/6 mice were used for this study; mice classed as young (8–12 weeks of age), multiparous (Dam) mice (which have previously given birth to litters), and aged mice (12 months of age). Aged mice were purchased from the Animal Resource Centre (*ARC*; Perth, Australia) at 12 weeks of age and were housed at Griffith University Animal Facilities for a further 9 months. Dam mice were purchased from ARC as ex-breeder mice and were between 11–12 months of age. Urine was collected 24 h prior to challenge and examined microscopically and by culture to exclude mice with a pre-existing condition, as described elsewhere [25]. Mice received food and water *ad libitum* and all procedures were approved by the Griffith University Animal Ethics Committee (approval number: MSC/03/12/AEC). The animals were monitored daily during the experimental procedure. A protocol was used to define early/humane endpoints in cases where mice might become severely ill prior to the experimental endpoint; this included escalated monitoring of mice based on clinical signs (locomotion, behaviour, appearance), which were evaluated using a numerical score-sheet designed in consultation with a veterinarian to determine when to euthanize the mice.

The murine model of UTI, utilizing female C57BL/6 mice and transurethral inoculation was used, essentially as described elsewhere [11, 22]. Briefly, mice were challenged with 40–50 μl of PBS containing 10^9^ cfu of UPSA 0247 using a sterile Teflon coated catheter (0.28 mm internal diameter, 0.61 mm outer diameter, 25 mm length; Terumo, Somerset, NJ) inserted directly into the bladder. Control mice received sterile PBS. Methods used to alleviate suffering/distress included anesthesia during the challenge procedure (isoflurane, 3–4% atmosphere), minimal handling and housing to include environmental enrichment. Urine was collected at 24 and 48 h post infection (p.i.) and used for colony counts. Separate groups of mice were euthanized at 24 and 48 h p.i. and the bladders were aseptically removed and processed for colony counts or used for 23-target multiplex protein assays (BioPlex, *BioRad*). The method of euthanasia was cervical dislocation. For multiplex protein assays, bladders were homogenised in PBS containing cOmplete™ ULTRA protease inhibitor (*Roche*). Each experiment used at least 8 mice per group and all experiments were performed twice, independently over a period of 3 years.

### Measurement of soluble inflammatory mediators

Bladder supernatants (50 μl) prepared from groups of PBS control and infected mice were used to quantitate 23 cytokines in the tissue in response to infection. We used a mouse multiplex protein assay kit for these measurements (*BioPlex*, *BioRad*), which were performed according to the manufacturers’ instructions.

### Statistical analyses

Colony count data derived from the analysis of bladders and urine were examined for normality using Shapiro-Wilk tests, and differences between groups were compared using Kruskal-Wallis One-way Analysis of Variance (ANOVA) tests, followed by Dunn’s multiple comparison post-tests, with significance set at *P* < 0.05. Cytokine expression data were compared using Kruskal-Wallis ANOVA, and specific pair-wise comparisons were performed using Mann-Whitney U tests with significance set at *P* < 0.05. Group sizes and replicates in experiments are indicated in each figure legend. All of the statistical analyses were performed using GraphPad Prism version 6.0.

## Results

### Levels of *S*. *agalactiae* colonisation of bladder tissue are similar between young and aged mice but bacteriuria levels differ markedly between young, aged, and dam mice

**Fig 1:**
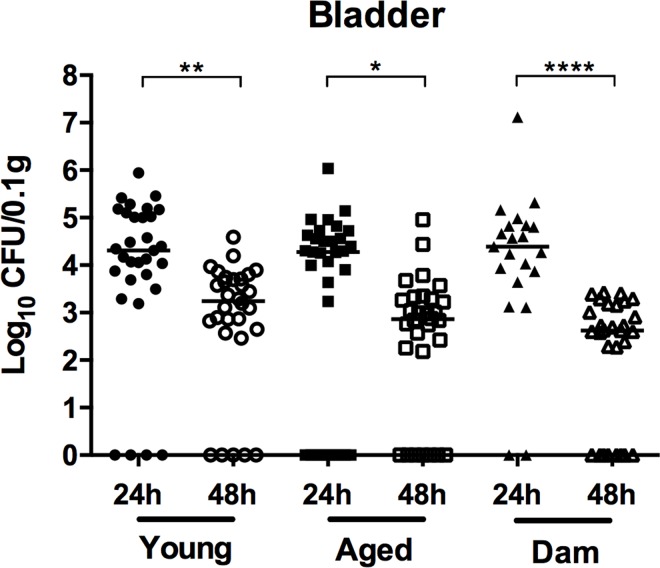
*Streptococcus agalactiae* bacterial loads in the bladder in young, aged, and dam C57BL/6 mice at 24 and 48 h post-infection. Data are a combination (n = 21–32) of 3 separate experiments each with 8–12 mice. Bars represent medians and are shown with interquartile ranges to illustrate the comparisons between groups based on Kruskal-Wallis ANOVA tests, and Dunn’s multiple comparison post-tests, with significance levels indicated (* *P* < 0.05; ** *P* < 0.01; **** *P* < 0.0001).

To determine whether age and/or parity affects the ability of *S*. *agalactiae* to colonize the urinary tract, young, aged, and dam mice were challenged with UPSA 0247 and bacterial titres in the bladder and urine were examined at 24 h and 48 h p.i. We compared the numbers of bacteria recovered from the bladders of young, aged, and dam mice at each time point, which revealed significant decreases in the medians from 4.31, 4.27 and 4.39 at 24 h to 3.24, 2.86 and 2.62 log_10_ cfu 0.1g^-1^ bladder at 48 h, respectively (Fig 1). There were no significant differences in the bladder tissue bacterial burdens comparing the infected groups at 24 h and 48 h p.i. There were no instances of animals dying prior to the experimental endpoint.

**Fig 2:**
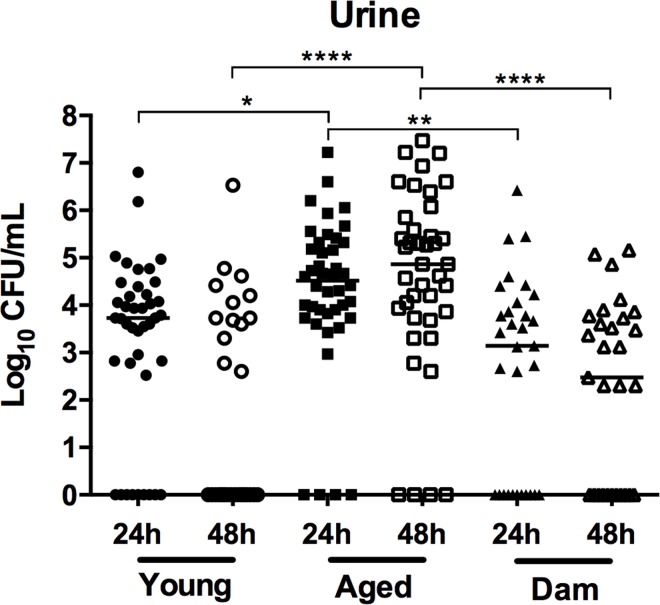
*Streptococcus agalactiae* bacterial loads in the urine in young, aged, and dam mice at 24 and 48 h post-infection. Data are a combination (n = 29–42) of 3 separate experiments each with 8–12 mice. Bars represent medians and are shown with interquartile ranges to illustrate the comparisons between groups based on Kruskal-Wallis ANOVA tests, and Dunn’s multiple comparison post-tests, with significance levels indicated (* *P* < 0.05; ** *P* < 0.01; **** *P* < 0.0001).

The numbers of *S*. *agalactiae* recovered from the urine of young mice were also significantly different between all groups according to Kruskal-Wallis ANOVA tests. The median bacteriuria loads in aged mice (4.52 and 4.86 at 24 h and 48 h p.i., respectively), dam mice (3.15 and 2.48 at 24 h and 48 h p.i., respectively) and young mice (3.73 and 0 at 24 h and 48 h p.i, respectively) did not significantly differ between the 24 h and 48 h time points (Fig 2). Strikingly, we observed that aged mice exhibited significantly higher bacteriuria loads than young mice (*P* < 0.01, *P* < 0.0001; Fig 2), and dam mice (*P* < 0.0001, *P* < 0.0001; Fig 2), at 24 h and 48 h p.i., respectively. Thus, aged mice are significantly restricted in their ability to clear bacteriuria due to *S*. *agalactiae* compared to young mice and dam mice, and multiparity is associated with significantly better clearance of bacteriuria due to *S*. *agalactiae* compared to age-matched nulliparous mice.

We also analysed bacterial loads in the kidneys of mice and, consistent with published data showing that colonisation occurs at a low frequency in C57BL/6 mice [26–28], we recovered bacteria from the kidneys in ≤ 10% of the mice in each group (data not shown). Together, these data establish that increased age, but not parity is associated with a higher bacteriuria burden due to *S*. *agalactiae* but tissue bacterial loads are similar between young mice and aged mice.

### Cytokine production in the bladder following *S*. *agalactiae* UTI differs between young and aged mice and changes over time

**Fig 3:**
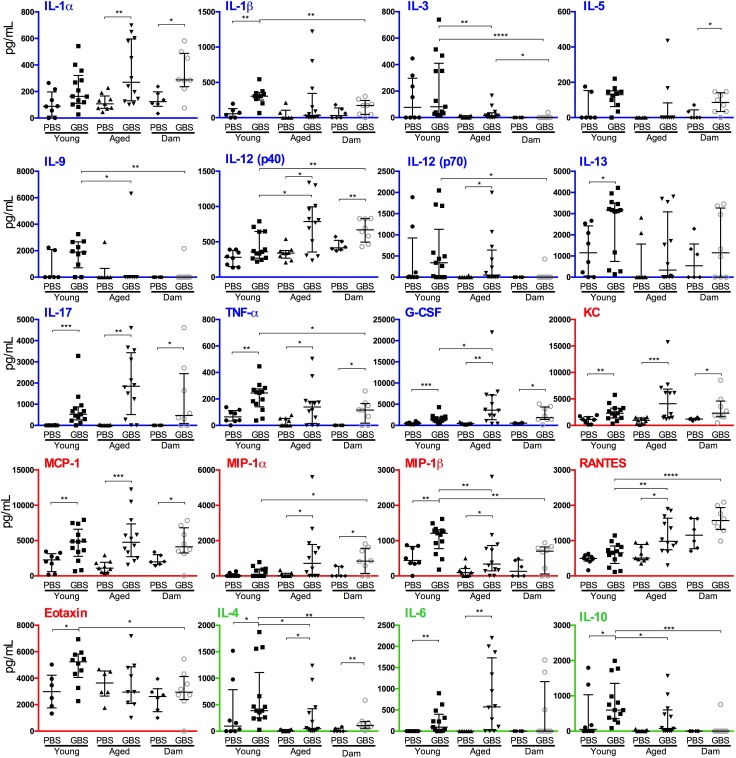
*Streptococcus agalactiae* UTI induces distinctive patterns of cytokine secretion in young, aged, and dam mice at 24 h post-infection. Pro-inflammatory cytokines and growth factors (blue), chemokines (red) and regulatory cytokines (green) are highlighted using coloured axes. Median bars and interquartile ranges are shown for 3 independent experiments combined (n = 6–13). The data were initially compared using Kruskal-Wallis ANOVA, and Dunn’s multiple comparison post-tests. Additional pair-wise comparisons were performed using Mann-Whitney U tests to compare PBS and infected conditions for cytokines across the same host background. Significance levels for the latter are indicated (* *P* < 0.05; ** *P* < 0.01; *** *P* < 0.001; **** *P* < 0.0001). The figure denotes *S*. *agalactiae* as group B streptococcus (GBS).

Analysis of cytokines identified major differences in the bladder immune response to infection in young, aged, and multiparous backgrounds. Initial multiple comparisons revealed many significant differences between and within groups, particularly for IL-1β, IL-12(p40), MCP-1, IL-17 and others. We subsequently performed pairwise comparisons using Mann-Whitney U tests to focus on comparisons of i) infected and control mice within a host background (i.e. Young, Aged, Dam), and ii) infected conditions across different host backgrounds. This analysis revealed significantly higher levels of several cytokines in aged and young mice following infection (compared to PBS controls) at 24 h p.i., including IL-6 and MIP-1β, which were not significantly elevated in dam mice (Fig 3). We also identified differences between the responses of young and aged mice to infection, including four markers that were significantly elevated in young mice (IL-1β, IL-10, IL-13, eotaxin) but not aged or dam mice. In aged mice, five markers were significantly elevated following infection, but these were not significant in young mice (IL-1α, IL-12(p40), IL-12(p70), MIP-1α, RANTES) (Fig 3). The levels of IL-3, IL-4, IL-9, IL-10 and MIP-1β were significantly reduced in infected aged mice when directly compared to infected young mice, and IL-12(p40), RANTES and G-CSF were increased (Fig 3). A synopsis of the *P* values derived from multiple comparisons and pair-wise comparisons summarizing the consistency in significance for several factors and discordance for some other cytokines (i.e. *P* > 0.05 from multiple comparison tests vs. *P* < 0.05 from pair-wise comparisons), is shown in S1 Fig.

**Fig 4:**
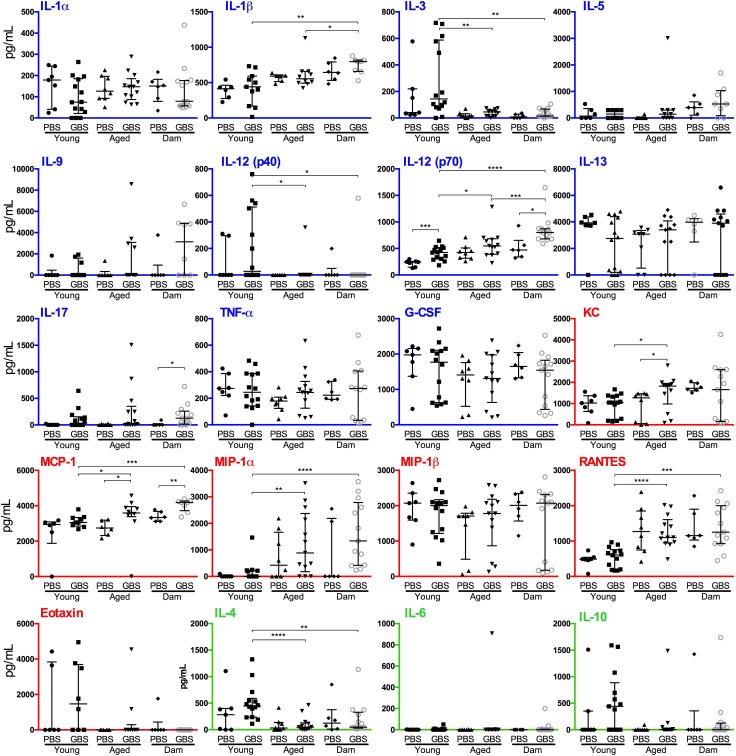
*Streptococcus agalactiae* UTI induces distinctive patterns of cytokine secretion in young, aged, and dam mice at 48 h post-infection. Pro-inflammatory cytokines and growth factors (blue), chemokines (red) and regulatory cytokines (green) are highlighted using coloured axes. Median bars and interquartile ranges are shown for 3 independent experiments combined (n = 6–13). The data were initially compared using Kruskal-Wallis ANOVA, and Dunn’s multiple comparison post-tests. Additional pair-wise comparisons were performed using Mann-Whitney U tests to compare PBS and infected conditions for cytokines across the same host background. Significance levels for the latter are indicated (* *P* < 0.05; ** *P* < 0.01; *** *P* < 0.001; **** *P* < 0.0001). The figure denotes *S*. *agalactiae* as group B streptococcus (GBS).

At the 48 h time point we observed fewer significant responses to infection overall, with elevations in the levels of IL-12(p40) in young mice, and KC and MCP-1 in aged mice (Fig 4) compared to their respective controls. There were no factors with significant differences shared among aged and young mice at 48 h p.i. Consistent with the observations of infected young and aged mice at 24 h p.i., there were significantly lower levels of IL-3 and IL-4, and higher levels of IL-12(p40) and RANTES in infected aged mice at 48 h p.i. (Fig 4 and S1 Fig). In addition to these, there were elevated levels of IL-12(p70) and reduced levels of KC, MCP-1, and MIP-1α in the infected aged mice compared to the young group.

### Multiparity alters the early bladder cytokine response to *S*. *agalactiae* infection

Comparison of the immune response in multiparous dam mice and nulliparous aged mice (age-matched at 11–12 months of age) at 24 h p.i. identified significantly higher levels of several cytokines due to infection and dependent on the host background. For example, infected dam mice exhibited higher levels IL-1α, IL-4, IL-12(p40), IL-17, G-CSF, KC, MCP-1, MIP-1α and TNF-α compared to age-matched PBS-controls (Fig 3). There were also higher levels of IL-5 in dam mice that were not significant in aged mice. Comparing infected dam mice and infected aged mice at 24 h p.i. revealed only IL-3 as significantly elevated in the latter group.

There were significant differences in the levels of only a few factors between infected multiparous mice and PBS-control mice at 48 h p.i., including elevated levels of MCP-1, (consistent with aged mice), IL-17 and IL-12 (p40). In comparing the response of infected aged and infected dam mice, we observed significantly higher levels of IL-1β and IL-12(p40) in dam mice (Fig 4).

### Comparative immune responses to infection between mice groups and time points

The immune responses that were consistent across all three infected mice groups at the early (24 h) time point included IL-17, G-CSF, TNF-α, IL-4, KC and MCP-1 (S1 Fig [part C], Fig 3). Thus, these factors are produced in response to infection in this model, independent of age and parity. The responses that were consistent among aged mice and (age-matched) dam mice (but not significant in young mice) included IL-1α, IL-12(p40) and MIP-1α. Cytokines that were significantly elevated in nulliparous mice (young and aged) but not dam mice were IL-6 and MIP-1β (S1 Fig [part C], Fig 3). The cytokine profiles of infected young mice showed no clear similarities between the 24 h and 48 h time points. In aged mice, KC and MCP-1 had elevated levels at both 24 h and 48 h, the latter of which was also elevated in dam mice, together with IL-17 and IL-12(p40) (S1 Fig [part C], Fig 3). Comparing the responses of infected young mice to either aged or dam mice, identifies IL-3, IL-4, IL-9, IL-10 and MIP-1β as significantly elevated in young mice, and RANTES, G-CSF, and IL-12(p40) as significantly elevated in the aged mouse groups at 24 h (S1 Fig [part D], Fig 4). At 48 h, IL-3, IL-4 and IL-12(p70) are elevated in young mice compared to either aged or dam mice, and IL12(p40), RANTES, MIP1α and MCP-1 are elevated in the aged and dam mice compared to young (S1 Fig [part D]). Thus, comparative profiling of bladder immune responses to *S*. *agalactiae* infection shows significant effects of age and parity towards a number of well-described chemotactic, pro-inflammatory and regulatory cytokines.

## Discussion

Bacterial UTIs cause massive economic, societal and healthcare costs and are among the most frequently occurring infections in humans. *S*. *agalactiae* has emerged in recent years as an important gram-positive aetiological agent of UTI [7, 10]. During the past decade, while rates of neonatal *S*. *agalactiae* disease have declined [29], the burden of *S*. *agalactiae* disease in elderly adults has increased and UTIs are among the infections that are increasingly recognized [7, 10]. The current study aimed to determine whether host background, in the context of age and parity, might affect the pathogenesis of UTI, including the level of colonization of and local immune response to *S*. *agalactiae* in experimentally-infected mice. One study that examined differences between aged and young mice with *S*. *agalactiae* UTI [24] reported lower bladder colonisation in aged mice vs. young mice, and higher colonisation with parity [24]. The results of the current study show (i) equivalent levels of bladder tissue colonisation in young and aged mice, in contrast to prior findings [24], (ii) equivalent levels of bladder tissue colonisation in young and dam mice, consistent with [24], (iii) increased age, but not parity predisposes to higher bacteriuria burdens during *S*. *agalactiae* UTI, and (iv) both age and parity effect the patterns of production of several regulatory and pro-inflammatory cytokines, including KC, MCP-1, IL-17, MIP-1α and RANTES that are expressed during *S*. *agalactiae* UTI in mice. Together, these findings provide essential novel insight into the significant role that host background, viz. age and parity, plays in determining the course of experimental UTI due to this important human pathogen.

One of the most notable findings of the current study is the effect of increased age towards the severity of *S*. *agalactiae* bacteriuria. Bacteriuria is a central aspect of UTI and can be important in the context of ABU and persistent colonization of urine [15, 30, 31]; bacteriuria was not studied in a prior report of *S*. *agalactiae* UTI in aged mice [24]. In a clinical context, studies have shown higher prevalence rates of bacteriuria in elderly populations, as reviewed elsewhere [18]. Unequal abilities of bacteria to grow in urine are known [32, 33], and were recently reported for *S*. *agalactiae* [15]. Understanding the nature of bacteriuria for clinical applications of novel therapeutic approaches, and to define the coevolution of hosts and bacteria, is emerging as a critical area [34–36]. Higher titre *S*. *agalactiae* bacteriuria in aged mice compared to young mice, and an inability of aged mice to reduce bacteriuria in contrast to young mice provide evidence of heightened susceptibility to *S*. *agalactiae* bacteriuria with increasing age.

The increased susceptibility of elderly individuals to UTIs is a multifactorial process and is thought to encompass weakening of the bladder muscles or other abnormal bladder function, bladder outlet obstruction, increased use of long-term indwelling catheters, alterations in hormonal, metabolic and immune function, and increased use of various medications that may impact UTI [3, 18, 37]. Other immune dysregulation, rather than impairment of immune system function, has been associated with the effect of increased age towards impaired ability to respond to and eradicate microbes [38]. The rates of eradication of UTI with any duration of antibiotic therapy are lower in elderly adults compared to younger women [39], and there is the emergent issue of overtreatment with antibiotics for suspected UTI in elderly adults that leads to negative consequences such as multidrug-resistant organisms, as reviewed elsewhere [40]. Thus, an improved understanding of the nature of heightened susceptibility to bacteriuria in elderly adults, and *S*. *agalactiae* specifically, as observed in this study, is an important goal for future studies. Recent findings of changes in metabolic characteristics and urine composition coincident with increasing age [41–44] suggest that comparisons of the ability of bacteria to grow in urine from young and aged individuals might provide interesting insight into the observed differences in bacteriuria noted in this study.

Due to the relationship between age and parity and UTI, we explored the effects of previous pregnancy towards infection and immune responses to *S*. *agalactiae* UTI by studying dam, age-matched nulliparous and young mice. A prior study reported on the susceptibility of multiparous mice to *S*. *agalactiae* single species UTI, polymicrobial UTI and ascending UTI due to UPEC [24]. In the current study dam mice exhibited equivalent bacteriuria compared to young mice but less bacteriuria than age-matched nulliparous mice. The effects of parity on UTI may differ for distinct uropathogens and mixed infections; however, the seemingly paradoxical new finding that, even though human pregnancy is associated with higher UTI risk, dam mice had less bacteriuria, suggests that parity somehow aids in controlling *S*. *agalactiae* bacteriuria. There is scant literature on urinary composition related to post-pregnancy that may explain this finding. It is possible that different urinary compositions between dam and age-matched nulliparous mice may affect bacterial growth in urine or other aspects of the host-pathogen interactions in the bladder. In one prior study, alterations in hormonal conditions and urinary tract anatomy was linked to the disproportionally high rates of UTIs among pregnant women [18]. In ovariectomized mice, which have been used as a model to study UTI in menopausal women, pathogenesis is subject to effects that are related to estrogen availability. Together, these observations highlight the important role that hormonal conditions might play in determining bladder health and pathogen defense [45]. The disparities observed in bacteriuria between age-matched dam and nulliparous mice in the current study are thus of interest for future studies of any potential effects of urinary composition and/or hormonal changes in this model.

Comparing immune responses of young and aged mice to *S*. *agalactiae* UTI shows that aged mice produce several cytokines in the bladder in a similar fashion to young mice at 24 h following infection. In contrast, aged mice produce more IL-12(p40), G-CSF and RANTES than young mice, and less IL-3, IL-9, IL-4, IL-10 and MIP-1β. The observations of elevated levels of G-CSF and RANTES in aged mice in the current study support prior reports that have shown increased levels of other chemokines in aged populations [46–48]. The current study also identifies several factors, including IL-1α, MIP-1α, RANTES, IL-12(p40) and IL-12(p70) that are induced in aged mice but not young mice following infection (compared to PBS) and other factors, including IL-1β, IL-10, IL-13 and eotaxin that are specific to young mice. The observations of elevated levels of IL-1β in young mice in the current study are consistent with a prior report that showed younger adults produced higher levels of IL-1β compared to elderly individuals [46]. However, in contrast to prior studies that report high levels of IL-6 in elderly adults compared to younger adults [46, 49, 50], we failed to detect a statistically significant difference in the levels of IL-6 between young mice and aged mice in the current study (medians: young 95 pg/mL vs. aged 572 pg/mL, *P* = 0.11; Fig 3). Taken together, the observations of the current study pin-point several cytokine responses that are consistent with prior literature and other responses that are unique to our study. Finally, the cytokine responses observed in the bladders of mice at 24 h subsided by 48 h, at which time, and in a similar way to the observations of cytokine responses in young and aged mice, there were few factors showing differential levels between infected and PBS control mice.

It is important to consider the observations of cytokine responses in the current study in the context of an expansive literature on “immune system aging” and the changes that occur in the immune system with increased age, termed immunosenescence [51, 52]. The literature offers insight into the wide-ranging changes in cytokine production that can occur with increased age and the complexity and nuances that can be associated with such changes. Importantly, the impracticality of generalizing cytokine responses during immunosenescence reflects (i) distinct effects in the innate and adaptive arms of the immune response [52], (ii) dissimilarities between cell types and immune system compartments [51], (iii) unique study designs that include species-specific differences, and potential assay artefacts such as plastic-ware effects [53], and (iv) animal models that are limited in translational capacity to human biology [54]. Collectively, these issues can constrain comparisons of individual studies and warrant an avoidance of endeavouring to oversimplify the cytokine data in the current study.

Adding to the aforementioned complexity of the literature on immunosenescence are conflicting reports on the effects of increased age towards cytokine responses in young and aged cells. For example, one study reported increased levels of pro-inflammatory cytokines, including IL-6 and TNF-α in some types of aged cells (e.g. B cells and dendritic cells), and plasma, as reviewed elsewhere [55]. These findings are consistent with changes in the T cell compartment during aging that include increased expression of pro-inflammatory cytokines and IL-6 [51]. In contrast, another study reported no difference in pro-inflammatory cytokine secretion between young and aged cells, including in dendritic cells [56]. In addition, studies of rodents have reported a decline in pro-inflammatory cytokine production, including for IL-6 and TNF-α with aging [52]. Apparent contradictions such as these should be viewed in the context of the broader immunosenescence literature, as discussed above, and, in particular, the changes in cytokine expression that can be cell type-dependent, such as for effector lymphocytes [51], and affected by study design. Finally, to what extent differences in the degrees and patterns of cytokine production between young and aged conditions are influenced by the type of microbial challenge or species differences, remains unclear.

It is of note that we measured cytokine levels in bladder tissue from mice but we did not measure cytokines in urine in the current study, which would be of interest given our finding that increased age predisposes mice to a higher burden of *S*. *agalactiae* bacteriuria. Future studies to examine cellular changes in response to infection, including inflammatory infiltrates in the bladder and neutrophil levels in urine sediments would also provide a more complete picture of cytokine release and cytological responses in this model. It is noteworthy that the type of innate immune response that occurs during *S*. *agalactiae* UTI is pathogen-specific [12]; and it would thus be of interest to investigate the effects of age and parity on UTI caused by other bacterial uropathogens to explore potential differences in innate immune responses triggered by distinct pathogens. It is also noteworthy that we used a *S*. *agalactiae* strain originally isolated from a case of uncomplicated acute cystitis [12]. Differences in the early steps in pathogenesis of *S*. *agalactiae* UTI have been reported related to strain differences [14]; some of these differences may relate to virulence factors such as the hemolysin [14, 57], or capsule, the sialic acids of which enhance the survival of *S*. *agalactiae* in the urinary tract [13] and augment the ability of *E*. *coli* to survive in the bladder [21]. The inoculum dose also affects bladder colonization in mice, according to escalating challenge dose studies [12]. Differences such as these may account for distinct observations related to the effects of age towards bladder colonization in mice in the current study, where no difference was detected in contrast to a previous study [24]. The overall prevalence of positive kidney cultures detected in the current study is also low compared to [24] but is largely consistent with prior reports of low rates of kidney colonisation following a single transurethral challenge in C57BL/6 mice [26, 27].

In summary, this study shows that the host environment plays an important role in influencing the severity of UTI due to *S*. *agalactiae*; infection dynamics, particularly in the context of bacteriuria, depend on age and parity, which also affect the nature of innate immune responses to infection. We conclude that UTI due to *S*. *agalactiae* in young, aged, and dam mice involves equivalent levels of bladder tissue colonisation in young, aged, and dam mice, but higher bacteriuria is associated with increased age but not parity. Both age and parity effect the expression of several key regulatory and pro-inflammatory cytokines and this may play a role in shaping the pathogenesis of these infections in different host backgrounds.

## Supporting Information

S1 Fig Comparative analysis of bladder immune responses of young, aged, and dam mice following *S*. *agalactiae* infection. Cytokine levels were initially compared using Kruskal-Wallis ANOVA and Dunn’s multiple comparison post-tests (A and B), followed by pair-wise comparisons of specific cytokines within host background groups across treatments (PBS control vs. infected) using Mann-Whitney U tests (C and D) with significance set at *P* < 0.05. Cytokines that showed significantly elevated levels in infected young, aged, or dam mice, compared to their respective PBS-mock infection controls, at 24 h and 48 h p.i., are highlighted with grey squares (A and C). Cytokines that showed significantly different levels between infected young and aged, young and dam, or dam and aged mice are also shown, with filled coloured squares indicating higher cytokine responses (B and D). Consistent responses between the 24 h and 48 h p.i time points are indicated with stars.
(TIF)
