# Supplementary figures and images for: Increased Age, but Not Parity Predisposes to Higher Bacteriuria Burdens Due to Streptococcus Urinary Tract Infection and Influences Bladder Cytokine Responses, Which Develop Independent of Tissue Bacterial Loads
Source: PLoS One. 2016 Dec 9;11(12):e0167732. doi: 10.1371/journal.pone.0167732 (PMC5147962; doi:10.1371/journal.pone.0167732)

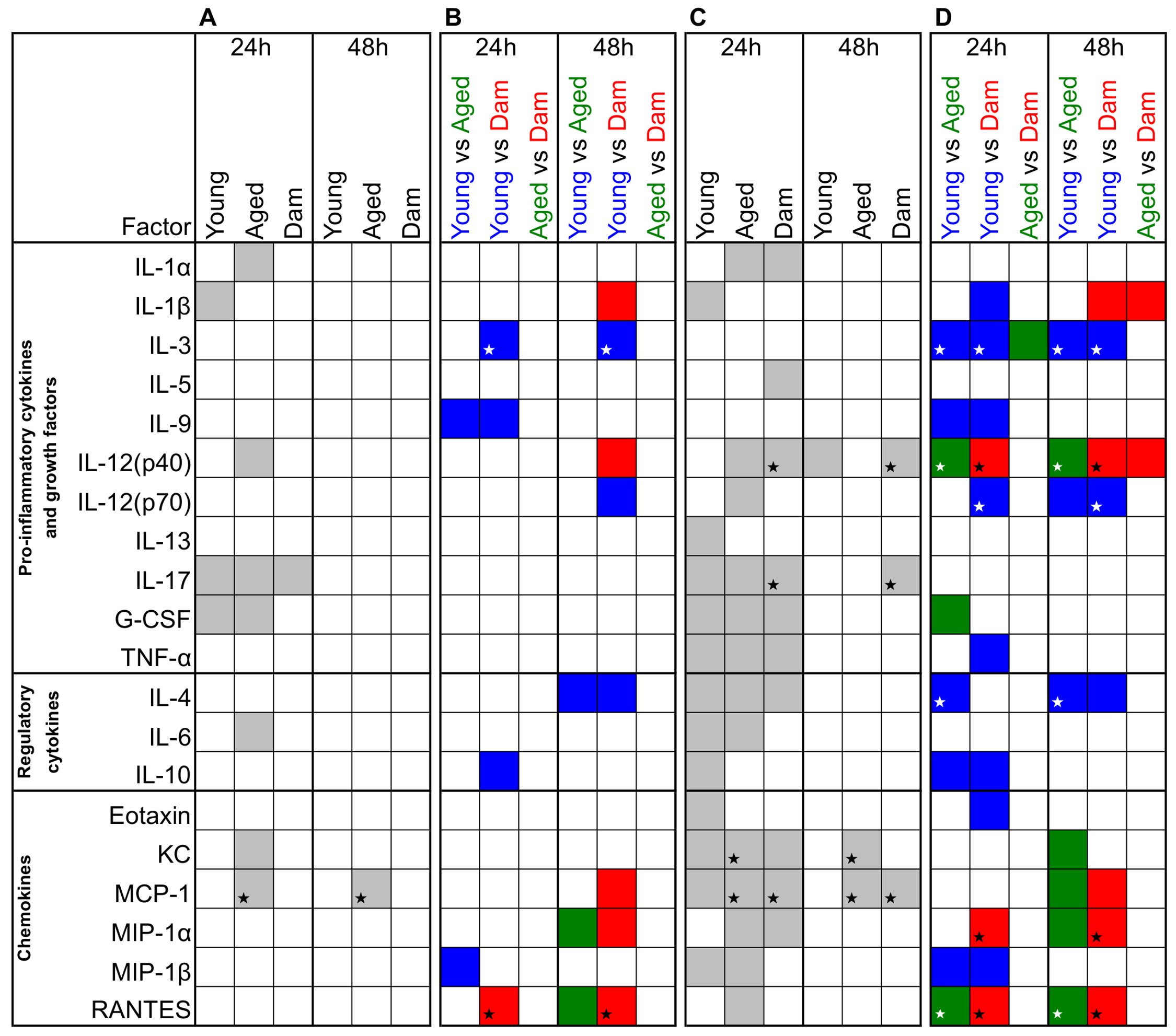

Supplement: S1 Fig — Cytokine levels were initially compared using Kruskal-Wallis ANOVA and Dunn’s multiple comparison post-tests (A and B), followed by pair-wise comparisons of specific cytokines within host background groups across treatments (PBS control vs. infected) using Mann-Whitney U tests (C and D) with significance set at P < 0.05. Cytokines that showed significantly elevated levels in infected young, aged, or dam mice, compared to their respective PBS-mock infection controls, at 24 h and 48 h p.i., are highlighted with grey squares (A and C). Cytokines that showed significantly different levels between infected young and aged, young and dam, or dam and aged mice are also shown, with filled coloured squares indicating higher cytokine responses (B and D). Consistent responses between the 24 h and 48 h p.i time points are indicated with stars. (TIF) [file pone.0167732.s001.tif]
